# Editorial: Community series in hepatic immune response underlying liver cirrhosis and portal hypertension, volume II

**DOI:** 10.3389/fimmu.2023.1305666

**Published:** 2023-10-11

**Authors:** Tian Lan, Sheyu Li, Haopeng Yu, Enis Kostallari, Jinhang Gao

**Affiliations:** ^1^Laboratory of Gastroenterology and Hepatology, State Key Laboratory of Biotherapy, West China Hospital, Sichuan University, Chengdu, China; ^2^Department of Gastroenterology, West China Hospital, Sichuan University, Chengdu, China; ^3^Department of Endocrinology and Metabolism, West China Hospital, Sichuan University, Chengdu, China; ^4^West China Biomedical Big Data Center, West China Hospital/West China School of Medicine, Sichuan University, Chengdu, China; ^5^Division of Gastroenterology and Hepatology, Mayo Clinic, Rochester, MN, United States

**Keywords:** liver immune microenvironment, liver cirrhosis, macrophage, immune response, mesenchymal stem cell, gut-liver axis

## Introduction

The immune landscape of a healthy liver plays an important role in maintaining tissue homeostasis. However, when subjected to injury, such as metabolic disorders, alcohol consumption, hepatitis viruses infection, or autoimmune diseases, the hepatic immunologic equilibrium is impaired, leading to tissue inflammation and fibrosis and culminating with cirrhosis and liver cancer (Yi et al.; Zhang et al.) (1). Although the immune response during liver disease has received significant attention, the detailed mechanisms by which residual and infiltrating immune cells interact with the liver parenchyma and contribute to liver disease initiation and progression remain to be further explored.

This Research Topic includes 13 articles and reviews that focus on the role of immune response in different types of liver injury and summarize the current knowledge on liver immunology and pathophysiology, as well as emerging therapeutic targets.

## Liver diseases and immune response

The immune response is a critical determinant in almost all kinds of liver diseases. Different types of immune cells actively participate in liver disease initiation and progression through various signaling pathways (Yi et al.; Zhang et al.) (2, 3). Several studies in this Research Topic have dissected distinct interactions between immune cells and the liver to facilitate the understanding of the underlying mechanisms in different liver pathological settings.

### Metabolic dysfunction-associated steatohepatitis

Metabolic dysfunction-associated steatotic liver disease (MASLD) is characterized by hepatocyte steatosis, metabolic disorders, and dysregulated hepatic immune microenvironment (Zhang et al.). The growing burden of MASLD is partly attributed to the increasing prevalence of obesity and diabetes (4, 5). Sphingosine 1-phosphate (S1P) is a bioactive lipid released by stressed hepatocytes. The S1P receptor is expressed in a wide range of immune cells and, by binding S1P, can induce immune cell infiltration into the liver and contribute to liver inflammation and MASH progression (6, 7). The inhibitor of S1P_1_, S1P_4_, and S1P_5_, etrasimod, showed a better effect than the single S1P_1_ inhibitor in reducing the proportion of pro-inflammatory infiltrating immune cells and inducing the expression of anti-inflammatory markers on macrophages (Liao et al.). Liver injury and inflammation in the MASH mouse model are ameliorated after etrasimod treatment (Liao et al.). These results suggest a potential therapeutic opportunity utilizing S1P inhibitors in patients.

### Chronic hepatitis B

Hepatitis B virus (HBV) infection is often chronic and difficult to eradicate because HBV can escape immune surveillance partly by impairing T-cell cytotoxic activity and cytokine production (8). CD8^+^ T cells from chronic hepatitis B patients or CD8^+^ T cells cocultured with hepatocytes infected with HBV have elevated expression of CD244, a regulator of immune functions (Xie et al.). Upregulation of lnc-AIFM2-1 and downregulation of miR-330-3p, two non-coding RNAs, induce the expression of CD244 on CD8^+^ T cells, contributing to the exhaustion of CD8^+^ T cells and subsequent HBV immune escape (Xie et al.). Non-coding RNAs have been indicated as potential contributors to liver diseases (9, 10). These findings further highlight the role of non-coding RNAs in the pathogenesis of chronic hepatitis B and propose novel therapeutic approaches by targeting HBV immune escape mechanisms.

### Autoimmune liver diseases

Autoimmune liver diseases comprise autoimmune hepatitis (AIH), primary sclerosing cholangitis (PSC), and primary biliary cirrhosis (PBC), which are highly associated with aberrant hepatic immune responses. In AIH, pro-inflammatory tetraspanin 1^+^ B cells are enriched in the liver and correlate with the severity of AIH. In addition, the CXCR3-CXCL10 pathway might be responsible for the recruitment of this B cell subgroup to the liver (Ou et al.). PBC and PSC are two major cholangiopathies where cholangiocyte functions are impaired. However, recent studies have unveiled a more active role of cholangiocytes in the pathogenesis of liver diseases. Cholangiocytes can dramatically switch their phenotype and secretory spectrum upon injury (Cai et al.). The soluble factors secreted by activated cholangiocytes, namely cholangiokines, have multifaceted effects on the liver, including the pro-regenerative, pro-inflammatory, pro-fibrotic, and pro-tumorigenic effects (11) (Cai et al.). The immune dysregulation affecting cholangiocytes is a key mechanism in PBC (Yang et al.). Nevertheless, the potential treatment options for PBC targeting the aberrant immune response are limited (12). Recently, encouraging results have been shown by targeting immune cells (such as the anti-CD20 monoclonal antibody rituximab targeting B cells) or chemokines (such as the dual CCR2/CCR5 inhibitor cenicriviroc) in patients with PBC or 2OA-BSA-induced PBC mouse models (Yang et al.) (13, 14). More clinical investigations are needed to evaluate the effect of novel therapies on autoimmune liver diseases.

### Decompensated cirrhosis

Decompensated liver cirrhosis is the advanced stage of liver cirrhosis with impaired hepatic and systemic immune responses, including immunosuppression (15). In the relatively early stage of liver cirrhosis, the replication of torque teno virus, a marker of immunosuppression, is already observed in patients (Rueschenbaum et al.). Moreover, a drop in the number of lymphocytes and a decline in T cell functions are found in patients with decompensated cirrhosis, which predict the development of acute-on-chronic liver failure (ACLF) (Rueschenbaum et al.). After ACLF is developed, while the abovementioned immunophenotype changes remain, additional changes, including neutrophilia with characterized neutrophil phenotype and increased macrophage M0-like monocytes, appear and further contribute to immunosuppression during ACLF (Weiss et al.). In decompensated cirrhotic patients with sepsis, myeloid-derived suppressor cells expand and exacerbate immunosuppression by boosting FOXP3+ T regulator cells and downregulating CD4^+^ T cell proliferation, which can be rescued by granulocyte-macrophage colony-stimulating factor (Sehgal et al.). Further studies are needed to understand the mechanism of immunosuppression during decompensated cirrhosis and explore novel therapeutic targets to prevent the progression toward ACLF.

## Emerging therapeutic approaches and targets

As the study of the immune response in liver disease has received increasing interest, potential novel anti-fibrotic or anti-cirrhotic therapies that target the hepatic immune microenvironment are being developed (Zhang et al.) (16). With the advance of regenerative medicine, mesenchymal stem cell (MSC) therapy has emerged as a promising treatment option for liver cirrhosis. MSCs are pluripotent stem cells capable of differentiating and replenishing the liver parenchyma (17). MSCs can also inhibit hepatic stellate cell activation and accelerate the degradation of extracellular matrix (Liu et al.; Yi et al.). Furthermore, the most appealing advantage of MSCs is that they can modulate the immune cell function through direct cell contact and paracrine signaling (including secretion of extracellular vesicles) (18, 19). MSCs can block the infiltration of pro-inflammatory immune cells while recruiting anti-inflammatory cells by secreting a wide spectrum of cytokines (Liu et al.; Yi et al.) (20, 21). Clinical trials have demonstrated promising outcomes of MSC therapy in patients with liver cirrhosis, showing benefits on the long-term survival rate and liver function (22, 23).

The gut-liver axis is another important area of investigation in the field of liver diseases. Gut-derived factors such as pathogen-associated molecular patterns, bile acids, and other metabolites can influence the composition of the liver immune microenvironment and contribute to liver disease progression (24, 25). Liver cirrhosis is often accompanied by gut microbiota dysbiosis, leading to the release of microbiota-specific factors. These factors can be sensed by Toll-like receptors (TLRs), a conserved family of pattern recognition receptors, triggering hepatic immune responses and influencing the progression of liver cirrhosis (Fan et al.). Targeting altered intestinal flora or TLRs, such as fecal microbial transplantation or inhibitors of TLR signaling, has been proposed as a potential treatment option for liver cirrhosis (Fan et al.) (26, 27). Some naturally occurring metabolites, such as neuropeptide galanin, also show a beneficial effect by alleviating liver inflammation and fibrosis in mice through modulating macrophage phenotype and function (He et al.). Further investigations are needed to identify the effect of these potential treatments in humans.

## Conclusion

This Research Topic highlights the role of the hepatic immune response in the progression of liver diseases. Emerging therapeutic targets and approaches for liver cirrhosis have been reviewed and discussed. Nevertheless, given the substantial heterogeneity within the cirrhotic niche (28), further research is imperative to enhance our understanding of the pathological mechanisms and discover effective treatment approaches for liver cirrhosis.

## Author contributions

TL: Writing – original draft, Writing – review & editing. SL: Writing – review & editing, Writing – original draft. HY: Writing – original draft, Writing – review & editing. EK: Writing – review & editing, Conceptualization, Funding acquisition, Supervision. JG: Conceptualization, Funding acquisition, Supervision, Writing – review & editing.
